# Homœopathy and Gynæcology

**Published:** 1883-08

**Authors:** 


Homœopathy and Gynæcology. By Thomas Skinner, M. D. Second edition. London: Homœopathic Publishing Company, 2 Finsbury Circus. 1883.

This interesting monograph of Dr. Skinner's is well known, and, we believe, well appreciated. Giving, as it does, the experience of one who has tried both allopathic methods and homœopathic therapeutics, this little work is a convincing argument in favor of pure Homœopathy. The work is *well* written, as are all of Dr. Skinner's publications.

